# Priority Areas for Large Mammal Conservation in Equatorial Guinea

**DOI:** 10.1371/journal.pone.0075024

**Published:** 2013-09-27

**Authors:** Mizuki Murai, Heidi Ruffler, Antoine Berlemont, Genevieve Campbell, Fidel Esono, Anthony Agbor, Domingo Mbomio, Agustín Ebana, Antonio Nze, Hjalmar S. Kühl

**Affiliations:** 1 Max Planck Institute for Evolutionary Anthropology (MPI-EVA), Leipzig, Germany; 2 Conservation International (CI), Arlington, Virginia, United States of America; 3 National Institute for Forestry Development and Protected Area Management (INDEFOR-AP), Bata, Equatorial Guinea; 4 National University of Equatorial Guinea (UNGE), Malabo, Equatorial Guinea; Università degli Studi di Napoli Federico II, Italy

## Abstract

Hunting is one of the main driving forces behind large mammal density distribution in many regions of the world. In tropical Africa, urban demand for bushmeat has been shown to dominate over subsistence hunting and its impact often overrides spatial-ecological species characteristics. To effectively protect remaining mammal populations the main factors that influence their distribution need to be integrated into conservation area prioritisation and management plans. This information has been lacking for Río Muni, Equatorial Guinea, as prior studies have been outdated or have not systematically covered the continental region of the country. In this study we evaluated: 1) the relative importance of local vs. commercial hunting; 2) wildlife density of protected vs. non-protected areas; and 3) the importance of ecological factors vs. human influence in driving mammal density distribution in Río Muni. We adopted a systematic countrywide line transect approach with particular focus on apes and elephants, but also including other mammal species. For analysis of field data we used generalised linear models with a set of predictor variables representing ecological conditions, anthropogenic pressure and protected areas. We estimate that there are currently 884 (437–1,789) elephants and 11,097 (8,719–13,592) chimpanzees and gorillas remaining in Río Muni. The results indicate strong hunting pressures on both local and commercial levels, with roads demonstrating a negative impact on elephants and overall mammal body mass. Protected areas played no role in determining any of the mammal species distributions and significant human hunting signs were found inside these protected areas, illustrating the lack of environmental law enforcement throughout the country. Río Muni is currently under-represented in conservation efforts in Western Equatorial Africa, and we recommend a focus on cross-boundary conservation, in particular in the Monte Alén-Monts de Cristal and Río Campo Ma’an conservation landscapes, where the highest densities and diversity of large mammals remain.

## Introduction

African tropical rainforests and the large mammals that inhabit them are today widely known for their increasing vulnerability in a progressively human-dominated environment [1]–[6]. Human population growth is occurring rapidly; in sub-Saharan Africa, the population is expected to grow at a rate of 2.5% per year compared to 1.2% in other continents [7]. Rising global demand for natural resources such as oil, wood and minerals, as well as for illegal wildlife products such as ivory and rhino horn, are putting increasing pressure on Africa’s remaining wildlife. In addition to the demand for resources by the United States and Europe, China has already had an astonishing impact on resource extraction and export in Africa [8]–[10], which is only set to increase as its economy continues to grow [9].

In several sites across the Congo Basin, the main form of human disturbance on many mammal species has been identified as hunting [11]–[13]. Commercial hunting in particular has been shown to be the principle driver behind bushmeat offtake [14] and is a stronger predictor of mammalian abundance and diversity than localised, subsistence hunting by villagers [15]. There are species-specific differences in the degree to which mammals exhibit negative correlations to intensified hunting, with larger-bodied species being the most vulnerable [16]. Generally speaking, hunting is exacerbated by recent development of logging roads that open up access into these forests [12], [17].

One of the conservation tools used to protect mammals that are affected negatively by logging and hunting is the allocation of protected areas (PAs) [18]–[22]. Proper management practices and law enforcement within these PAs are critical in ensuring their effectiveness, and without this they remain ‘paper parks’ [2], [23], [24]. It is important to note in addition, the significant conservation value of areas immediately outside of PA boundaries and the significant impact these areas can have on wildlife within the PA itself [25], [26].

We conducted this study across Río Muni, the mainland region of the Republic of Equatorial Guinea (EG), a country with a rapidly developing economy which is negatively impacting wildlife due to rapid infrastructure development and increased commercial hunting. Historically, the network of PAs has existed in EG by law since 1988 (Ley 8/1988) [20]. Today, PAs in Río Muni cover 15.4% of the country’s land yet there are limited law enforcement activities undertaken to manage and protect these areas since 2004 when ECOFAC (Conservation et Utilisation Rationelle des Ecosystèmes Forestieres en Afrique Centrale) suspended its activities in Monte Alén National Park (PNMA) [28], [29]. PNMA is the only PA that has received any effective protection since the creation of the PA network in Río Muni [30]. This lack of law enforcement has threatened the wildlife population, mainly through heavy commercial hunting [28], which has been facilitated in recent years by the expansion of the road network across Río Muni.

Several wildlife surveys have previously been conducted in EG. The first gorilla survey, conducted in 1966, provided a country density estimate of 0.58 to 0.86 gorilla per km^2^ following a survey of three main localities [31]. Subsequent survey work in 1989 led to an estimate of 0.22 to 0.45 nesting gorilla per km^2^, with the highest densities identified to be in Río Campo and the district of Nsork in the southeast of the country [32]. These data however, are now out-dated and furthermore, there have been some inconsistencies in the past findings. As for chimpanzees, no nationwide census had been made in the past; the best estimate - made only within the PNMA in 1994 - estimated a density of 5.35 nests per km surveyed [31]. Attempts at estimating elephant abundance in the last couple of decades had resulted in a range of estimated values from less than 500 individuals throughout the entire country [33] to 700 just within the southern part of PNMA [34]. The 2007 IUCN African elephant status report declared the elephant population in EG to be the least known in Central Africa [18].

Through this study we identify the current status of the Western lowland gorilla (*Gorilla gorilla gorilla*), Central chimpanzee (*Pan troglodytes troglodytes*), forest elephant (*Loxodonta cyclotis*) and other large mammal species (Table S1 [35]–[37]), and present the main threats that are influencing their distribution. In addition to obtaining an updated abundance estimate for apes and elephants, specifically, we address the following questions: 1) what is the impact of commercial vs. local hunting on large mammal distribution; 2) how effective are PAs in conserving large mammal populations; and 3) what is the importance of human vs. ecological impacts on large mammal distribution. This information is vital to understand the conservation requirements of Río Muni’s endangered mammalian flagship species, and to focus conservation efforts on priority areas to ensure the long-term protection of these remaining populations.

## Methods

### Study Area

**Figure 1:**
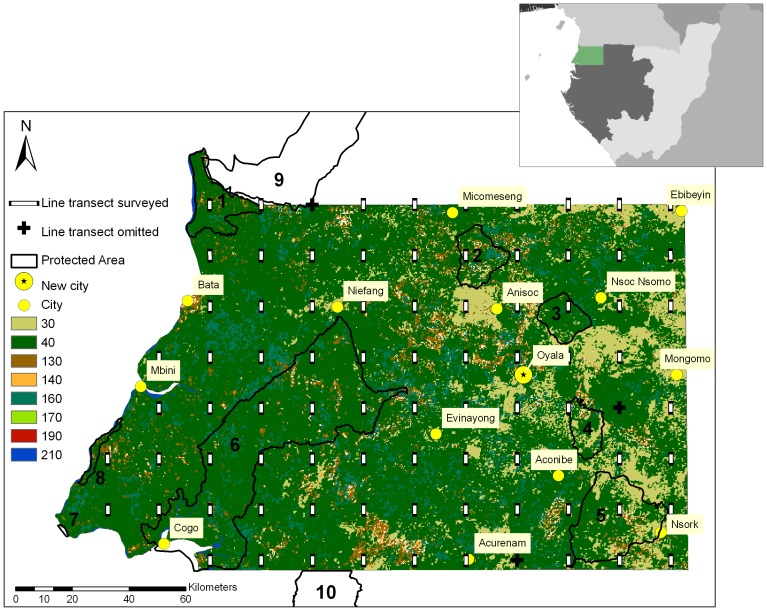
Río Muni landscape and study design. Inset illustrates the geographical location of Río Muni in Central Africa. Cities (yellow) across Río Muni and the new proposed capital city of EG, Oyala (yellow with star), are shown with the line transects surveyed (white bars) and transects that were not surveyed (black crosses). PAs: (1) Río Campo Nature Reserve; (2) Montes Temelón Nature Reserve; (3) Piedra Bere Natural Monument; (4) Piedras Nzas Natural Monument; (5) Altos de Nsork National Park; (6) Estuario del Muni Nature Reserve; (7) Playa Nendyi Scientific Reserve; (8) Punta Llende Nature Reserve; (9) Campo Ma’an National Park, Cameroon; (10) Monts de Cristal National Park, Gabon. Global cover layer shows, 30: Mosaic vegetation/croplands; 40: Closed to open broad-leaved evergreen or semi-deciduous forest; 130: Closed to open shrubland; 140: Closed to open grassland; 160: Closed to open broad-leaved forest regularly flooded; 170: Closed broad-leaved forest permanently flooded; 190: Artificial areas; 210: Water bodies.

Río Muni, the mainland of EG, lies between Cameroon and Gabon in western Central Africa and covers an area of 26,017 km^2^ (Figure 1). Vegetation is largely Guineo-Congolian rainforest, with mangrove forests in the southwest estuary, riparian palm forests along the coast and inselbergs commonly found in the east [32], [37]. Altitude reaches 1,113 m in the peak of PNMA, which is part of the Niefang Mountain range [31]. Human population is estimated at 700,401 across the whole of EG [38]. Average annual temperature is around 27°C and annual mean rainfall is 2,500 mm.

Forest concessions have grown significantly since the discovery of oil reserves in the early 1990s [39]. Between 1993 and 1997 concessions more than doubled to 1.5 million hectares, covering the entire commercially productive forest of the country [39]. The area further increased to 1.7 million hectares by 2001 [39]. Fuelled by EG’s oil boom, its economy has rapidly developed, with greater urbanisation and increased infrastructure development and road construction throughout Río Muni. Forest cover in EG is continuously on the decline and this decline has recently been accelerated with the governmental target of providing road access, water and electricity to every village by 2020 as part of the National Economic Development Plan: Horizon 2020 [38].

Furthermore, with increasing population and wealth and a lack of suitable alternative fresh protein source in EG, there is a large demand for fresh meat including bushmeat [40] amongst the urban elite and in particular within the dominant Fang ethnic group in Río Muni, whose diet largely comprises of meat [40], [41]. Studies have shown bushmeat hunting in EG to be at unsustainable levels [11], [40], [42].

### Field Methods

Permission to survey all PAs and non-PAs was provided by the National Institute for Forestry Development and Protected Area Management (INDEFOR-AP) of EG, and the Ministry of Agriculture and Forests. In addition, permission was gained from regional delegates of each of Río Muni’s districts and from the presidents of each village located near the transect starting points for access to their villages and the surrounding areas. This field study did not involve any direct contact with endangered or protected species. Furthermore, no animals were captured or handled during the study.

#### Survey design

We placed a systematic grid with random origin and cell size of 9×9 km across the country. Every 9×9 km cell was subdivided into nine smaller cells of 3×3 km. Transects of 3 km in length were then placed in the central 3×3 km cell of every second 9×9 km cell, oriented north-south. Thus the design contained 86 transects with a total length of 258 km (Figure 1).

#### General approach

For data collection we used standard distance line transect sampling [43]. Chimpanzees and gorillas were surveyed using nest counts, and elephants using dung counts. Other large mammal occurrences were recorded either by direct sightings or indirect signs of presence, such as vocalisations, dung or footprints (Table S1).

To convert ape nest and elephant dung counts into individual density, we estimated decay rates of these signs using the retrospective method proposed by Laing *et al*. 2003 [44]. Field staff were trained to these methods in a two-week workshop and two field trainings, each lasting four days, resulting in the selection of two teams of equal strength consisting of six members in each. All data were recorded on paper in the field and subsequently entered into a spread sheet after each field mission.

#### Estimating dung and nest decay rates

Nest and dung show spatio-temporal variability in decay rates [45]–[47]. In order to estimate study-specific decay rates of ape nests and elephant dung, a preliminary decay study using reconnaissance or ‘recce’ walks was conducted between March and mid-May 2011. During this decay study, we located freshly built chimpanzee and gorilla nests and fresh elephant dung at several locations across the country. We revisited each nest and dung once after a certain interval to note the degradation level, classified between 1 (fresh) to 5 (disappeared) [44], [48], [49]. In order to increase our sample size, we included fresh nests and elephant dung encountered during transect sampling into the decay study.

#### Line transects

For all transects, we hired one or two local villagers to create a linear route for the observing team members, as directed by a compass-bearer walking directly behind. The villager opened up the forest route with a machete, causing as minimal damage to the environment as possible. The compass-bearer, in addition to directing the cutter in a straight line, focused their attention to trees ahead for nests and direct wildlife observations. Several metres behind the compass-bearer was the data recorder with a GPS (Garmin 60CSx) to mark waypoints and to note down all observations on a data sheet. Observation was focused ahead, occasionally turning around to ensure no nests were missed above the line. Behind the data recorder, two observers, one on each side of the transect, walked in a zigzag manner within 5 m of the line, focusing particularly on nests that may be above the linear line. Finally the hip-chain bearer, walking behind the data recorder and in between the two observers, closed the team formation, recording the distance walked along each transect and focusing on observations of signs on the ground. Every member was also responsible for searching for any signs of mammal tracks, nests, dung, feeding remains, vocalisations, and direct sightings. Furthermore, human activities such as the presence of traps, shotgun cartridges, signs of logging, paths, roads and camps were recorded.

During transect sampling, field members communicated using hand gestures, or whispering only when necessary. Control waypoints were marked every 100 m on the GPS, based on the distance on the hip-chain. Hip-chains were tested every 500 m for 10 m with a tape measure to ensure accuracy in the readings. All changes in vegetation types, slopes and weather were additionally noted. Every observation documented had a GPS coordinate and the distance along the transect as determined with the hip-chain. For elephant dung and ape nests, perpendicular distance from the middle of the dung pile and from every nest to the line transect was recorded.

Ape nest decay stages were classified from 1 (fresh) to 4 (decayed) [50]. Classification class 5 was not used during line transects unlike in the decay survey as they would not be visible without prior knowledge of their existence. In the case of arboreal nests, data recordings include the tree species, diameter at breast height (DBH) and estimated overall height of the tree, and an estimated height of the nest within the tree. Where ground nests were found, the species of vegetation used for nest construction were recorded. Nest groups were identified using their decay stages within a distance of 50 m of each other. One challenge we faced was the discrimination of nests built by chimpanzees and gorillas. Existing methods for discriminating between nests of the two species require a large enough sample of nests assignable with certainty to either species [51]. However, in our study we did not find enough nests where we were sure of the builder’s species in order to apply such methods. Attributing the nests to chimpanzee or gorilla therefore relied on the experience and knowledge of the members of the teams. Generally, a group of arboreal nests were classed as being from chimpanzees and a group of ground nests from gorillas. However, this classification is likely to lead to overestimation of chimpanzee and underestimation of gorilla density (Text S1). We therefore refer to estimate based on tree nest only as maximum chimpanzee estimate and minimum gorilla estimate. Elephant dung piles were also classed from 1 (fresh) to 4 (decomposed) according to their decay stages [44], [52].

All transects were sampled from mid-May to the beginning of November 2011. Three transects could not be surveyed as two fell outside of EG (one in Gabon and one in Cameroon) and one was inaccessible. The start point of the inaccessible transect fell in the middle of Río Wele, one of the major rivers in EG, and we faced logistical constraints in reaching the other side.

### Analytical Methods

For data analysis we used design-based methods to estimate ape and elephant density. We used abundance and spatial model techniques to evaluate the importance of several predictor variables for apes, elephants and other large mammal species, and to also derive density distribution and countrywide population abundance values for apes and elephants. All abundance estimates were made for an area of 24,365 km^2^ as derived from available country GIS layers for Río Muni. This surface area differs from the aforementioned value that is generally cited, likely due to the effects of water courses.

#### Nest and dung decay rates

We estimated nest and dung mean decay time by applying the three models proposed by Laing *et al*. 2003 [44], which are the logistic model with left truncation, the logistic model with log transformed time variable, and the logistic model including an additional parameter for reciprocal time. Nest and dung decay classes were converted into binary format with classes one to four as ones and class five as zero (sign has disappeared completely and was not visible anymore). Inter-visit interval between first and second visit was calculated in days. We then fitted the three models to the data in R using the functions ‘glm’ for the ‘log’ model and ‘optim’ for the reciprocal and left truncation models [53]. We derived mean decay time by summing the products of daily decay probability and time elapsed since sign production over 10^4^ days. We obtained 95% confidence limits by bootstrapping the data 999 times [54]. To derive single decay time estimates for nests and dung, respectively, we calculated an Akaike Information Criterion (AIC) weighted mean of the different decay model estimates.

Chimpanzee and gorilla nests were combined to calculate the ape nest decay rate collectively as too few gorilla nests were found to make an independent estimate.

#### Design-based estimates

We estimated countrywide elephant and ape abundance using standard methods for obtaining sign detection functions for deriving the effective area covered along transects and conversion of signs into individual abundance by using Distance 6.0. Release 2 software [55]. We ran different combinations of key models and expansion terms to find the best fitting model that provided the lowest AIC (Table S2). All calculations were made according to Buckland *et al*. (2001) [43], and required auxiliary variables such as decay rate, sign production and proportion of nest builders, for which the latter two were taken from other literature (Table 1) [56]–[59]. Auxiliary variables for apes were calculated according to the weighted average of the proportion of chimpanzee and gorilla nests found.

**Table 1:** Values of auxiliary variables used for the calculation of chimpanzee, ape and elephant abundance [49].

	Chimpanzee	Gorilla	Ape^1^	Elephant
Proportion of nest-builders	0.83 [56]	0.77 [57]	0.82	–
Nest construction rate/dung production rate (per day)	1.09 [56], [58]	1.0 [58]	1.014	19.8 [59]

1 Values for apes were calculated as weighted averages based on the ratio of number of nests for the two species used in the analysis (chimpanzee: 323, gorilla: 51).

### Spatial Models

#### Covariates

Initially we selected 14 variables from the classes land cover, climate, topography, human impact and PAs (Table S2), to represent environmental conditions for wildlife in our models. The 14 predictor variables were partially correlated and also too many in proportion to the number of transects sampled and could therefore not directly be included into the analysis. We therefore conducted Principal Component Analysis (PCA) and Factor Analysis (FA) to condense the variables to a reduced number of predictors. However, neither PCA nor FA delivered a satisfying reduction of predictors by maintaining a high proportion of explained variance. We therefore selected ten of the variables (Table 2) representing the classes land cover, human impact and PAs of which no pair was highly correlated (Spearman correlation, max. *r*_s_ = 0.46; Table S3). Several of the predictors had skewed distributions and in order to achieve more symmetrical distribution we transformed these variables (Table 2).

**Table 2:** Predictor variables included in the GLM.

Variable	Resolution (m)	Description and metric used	Transformation	Source
Slope	1000	Mean slope in a circle centred on the transect midpoint with adiameter half the length of the transect	Log	CARPE
Roads	1000	Euclidean distance to all roads from midpoint of transect	Log	INDEFOR-AP
Agricultural mosaic habitat	311	Frequency of pixels classified as agricultural mosaic habitat (class 30of globecover dataset) in a circle centred on the transect midpointwith a diameter half the length of the transect	None	ESA 2009
Closed/open broad-leaved forest	311	Frequency of pixels classified as closed/open broad-leaved forest(class 40,160 of globecover dataset) in a circle centred on thetransect midpoint with a diameter half the length of the transect	None	ESA 2009
Settlements	1000	Euclidean distance to next settlement from midpoint of transect	Cube root	INDEFOR-AP
Cities	1000	Euclidean distance to cities from midpoint of transect	Square root	INDEFOR-AP
Distance inside PA	1000	Euclidean distance to PA border from midpoint of transect if withinPA and zero if outside of PA	None	CARPE

For web links to the data source see supporting information (Table S2).

### Response Variable

For calculating mammal richness, we used the total number of mammals detected and identified along each of the transects, based on direct and indirect observations.

For mammal body mass, we used available mammal body mass data [36], [60] and multiplied these values with the number of individuals per species encountered along each of the transects. These values were then summed per transect. We defined large mammals as being greater than 30 kg and medium mammals as those less than 30 kg, based on a subjective view of the available mammal body mass data (Table S1).

In order to distinguish the effects on different mammal groups, primate and ungulate richness were analysed separately (Table S1). Other groups could not be separated due to low detection rates.

### Modelling

To assess the combined influence of the six predictors and to evaluate our hypotheses, we applied Generalised Linear Modelling (GLM) [61]. We developed six models and ran them for chimpanzees, apes, elephants, mammal richness, all mammal body mass, large mammal richness, medium mammal richness, primate richness, ungulate richness and human signs. In order to build appropriate models and fulfil basic assumptions, we had to consider several issues. First, both ape nest and elephant dung count data are often skewed with a high proportion of transects with only few observations and few transects with a large number of nest or dung observations. We accounted for this by including a negative binomial error function into the models for chimpanzees and apes. Transect elephant dung data were too skewed, therefore we only used presence-absence information and a binomial error function. For a model evaluating mammal body mass we included a Gaussian error structure and for all other a Poisson error term. Second, our data were collected along transects of differing length. We therefore included an offset term into our model that related the response of the model to the counts on transects of differing length [62]. Third, to account for spatially autocorrelated residuals, we included an autocorrelation term as an additional predictor into the model (Text S2) [54]. Thus our full models became:

*response∼slope+distance to roads+closed/open broad-leaved forest+distance to settlements+distance to cities+distance inside protected areas+autocorrelation term+offset+error term*

To evaluate the combined influence of the six covariates we ran the full model. For reasons of model uncertainty in spatial model prediction, we also evaluated all possible combinations of models (*n* = 64) for which we derived AIC and AIC weights. All analyses were conducted using R [53].

In order to further understand the effect of ecological factors, we ran a second model where the distance to road variable was replaced by agricultural mosaic habitat. We had to ensure that none of the predictors were correlated, and hence both variables could not be used in the same model.

### Spatial Model Prediction

To predict ape and elephant density as well as mammal richness, mammal body mass and human sign distribution throughout the country, we first generated a grid with cell size of 1×1 km for the entire area of Río Muni (*n* = 24,365 cells). We then assigned the values of the six covariates to every grid cell (Table 3). Subsequently, we made predictions for every cell based on each of the 64 models fitted per response class. We then averaged the 64 predictions per grid cell and response class using the AIC weight for each model to derive a single value. For chimpanzees and elephants, we summed predictions for all cells to derive a nationwide abundance estimate. We derived 95% confidence limits by parametric bootstrap of model predictions.

**Table 3:** Results of nest and dung decay time estimates.

Species	Model	AIC^1^	AICw^2^	Decay time (days)	Lower CI^3^(days)	Upper CI^3^ (days)	Int^4^	Time	Logtime^5^	Rec time^6^
Chimpanzee	Left-truncated	52.4	0.49	149	130	160	4.35	−0.03		
	Log	53.3	0.31	167	132	266	16.42		−3.31	
	Reciprocal	54.2	0.17	149	127	181	4.47	−0.03		6.70
	Mean			**155**	**130**	**198**				
Ape	Left-truncated	69.6	0.53	153	126	202	2.60	−0.02		
	Log	72.7	0.11	262	145	646	8.72		−1.74	
	Reciprocal	70.3	0.36	148	124	182	3.60	−0.02		3.37
	Mean			**151**	**125**	**194**				
Elephant	Left-truncated	80.1	0.58	137	123	153	5.76	−0.04		
	Log	81.7	0.26	123	170	24	24.23		−4.97	
	Reciprocal	82.8	0.16	138	122	150	5.06	−0.04		7.24
	Mean			**138**	**123**	**157**				

1 Akaike information criterion;

2 Akaike information criterion weight;

3 Confidence interval;

4 Interval;

5 Logarithmic time;

6 Reciprocal time.

## Results

A survey effort of 233.94 km across 83 transects was completed. Four of the transects were terminated before the full 3 km length as part of each was outside of the country and seven of the transects were logistically impossible to complete due to obstacles that could not be navigated around, such as a cliff or mangroves.

### Nest and Dung Decay Rates

Twelve groups of chimpanzee nests, which totalled 76 individual fresh nests, were revisited after an interval ranging from 14 to 202 days from which a nest decay rate of 155 (130–198) days was calculated. Ape nest decay rate of 151 (125–194) days was calculated using 18 fresh gorilla nests revisited after 18 days in addition to the 76 tree nests.

108 fresh elephant dung were revisited on 11 occasions between 10 and 204 days to give a dung decay rate estimate of 138 (123–157) days.

### Apes

Out of the 83 line transects, chimpanzee nests were recorded along 51 of the transects, totalling a sum of 323 chimpanzee nests. These likely included some tree nests of gorillas which were misclassified. From these data, the Distance-based estimate provided a maximum chimpanzee population size of 6,418 (4,173–9,871) (Table S4; Figure S1). GLM prediction gave a maximum chimpanzee estimate of 7,824 (3,703–14,441) across Río Muni.

**Figure 2:**
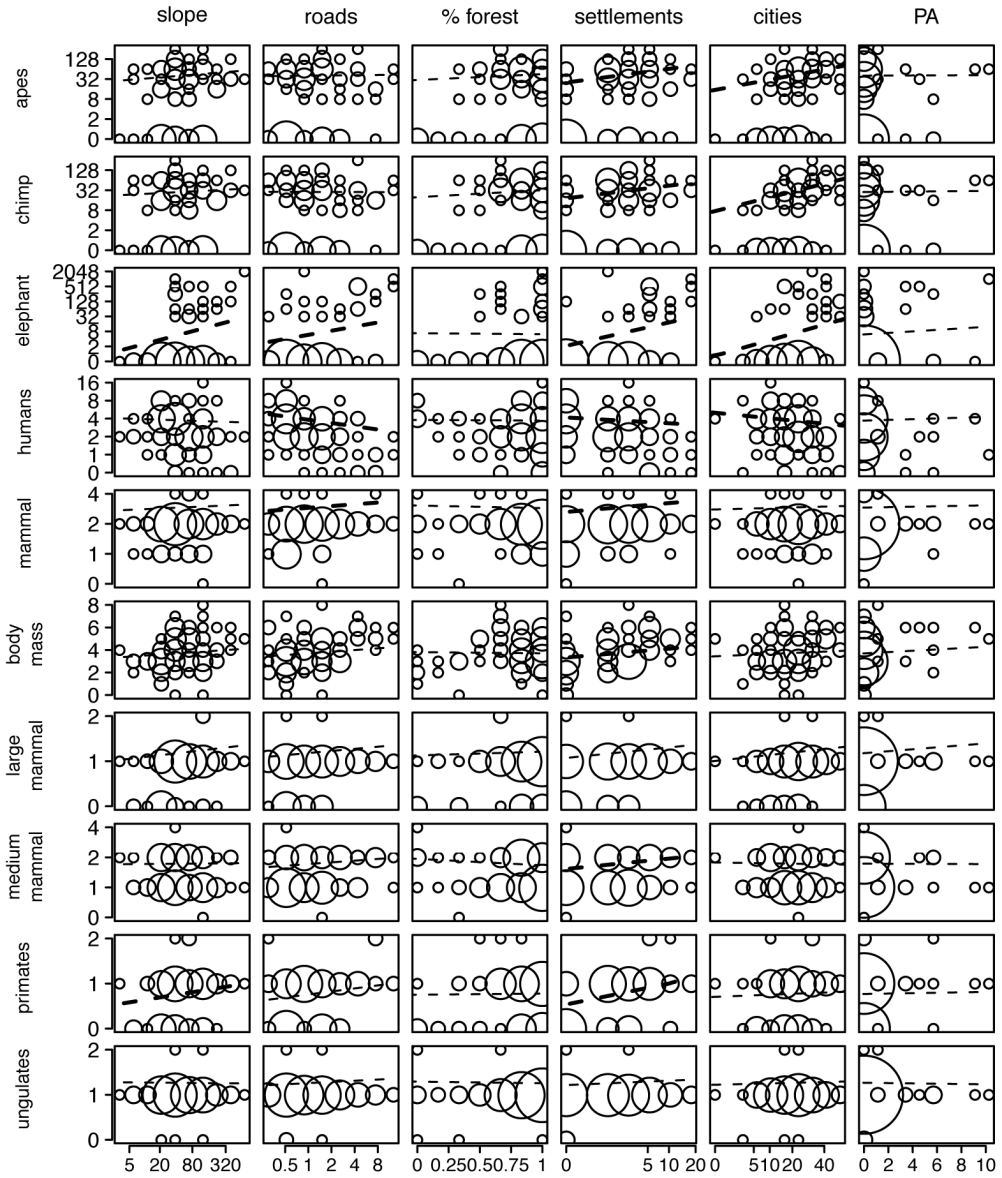
Plots of covariate effects. Observations (circles) and model predictions (lines) are shown for every combination of response and predictor. Transects are grouped and circle sizes are proportional to the number of transects. Bold lines represent significant effects. % forest signifies primary forest.

Proximity to cities and settlements were the only determining factors for chimpanzee abundance. Increasing distances from cities and settlements led to significant increases in chimpanzee abundance (Figure 2). Distance to roads and habitat type did not reveal any significance nor did slope nor PA (Table 4,5).

**Table 4:** AIC weighted average parameter estimates.

	Chimpanzee	Ape	Elephant	All mammalrichness	All mammalbody mass	Medium mammal richness	Large mammal richness	Primates	Ungulates	Human
Intercept			1.471	1.065	3.729	0.541	0.131	−0.254	0.183	1.282
Slope	0.036	0.132	1.076	0.016	0.177	0.000	0.044	0.110	−0.001	−0.012
Road	−0.024	−0.013	0.815	0.069	0.239	0.048	0.042	0.068	0.016	−0.320
Primary forest	0.045	0.022	−0.257	−0.017	−0.027	−0.061	0.010	−0.007	−0.003	0.005
Settlements	0.481	0.576	1.181	0.119	0.470	0.144	0.065	0.298	0.016	−0.077
Cities	0.762	0.542	1.136	0.004	0.051	−0.010	0.037	−0.004	0.005	−0.179
PA	0.000	−0.009	0.045	0.002	0.075	−0.005	0.022	−0.003	−0.002	0.040
AC term	0.249	0.499	−0.229	−0.012	0.293	−0.147	0.231	0.108	−0.056	0.294

**Table 5:** Full and null model results for each species and predictor variable.

	Chimpanzee		Ape		Elephant		All mammal richness		Mammal body mass	
Model	Full	Null	Full	Null	Full	Null	Full	Null	Full	Null
AIC	384.2	402.8	406.0	422.7	60.8	89.0	393.8	403.6	291.6	302.0
No. parameter	8	2	8	2	8	2	8	2	8	2
Intercept	3.18 (<**0.001**)	3.58 (<**0.001**)	3.56 (**<0.001**)	3.91 **(<0.001)**	1.32 (**0.03**)	2.43 (<**0.001**)	1.06 (<**0.001**)	1.08 (<**0.001**)	3.73 (<**0.001**)	3.73 (<**0.001**)
Slope	0.12 (0.46)	na	0.25 (0.10)	na	1.35 (**0.02**)	na	0.04 (0.30)	na	0.25 (0.12)	na
Road	-0.11 (0.48)	na	-0.08 (0.61)	na	0.95 (**0.04**)	na	0.08 (**0.04**)	na	0.30 (0.07)	na
Primary forest	0.11 (0.53)	na	0.04 (0.82)	na	-0.76 (0.22)	na	-0.05 (0.22)	na	-0.12 (0.49)	na
Settlement	0.54 (<**0.01**)	na	0.62 (**<0.001**)	na	1.34 (**0.03**)	na	0.12 (**0.01**)	na	0.44 (**0.02**)	na
City	0.73 (<**0.001**)	na	0.53 (**<0.001**)	na	1.39 (**0.01**)	na	0.01 (0.83)	na	0.10 (0.53)	na
PA	0.00 (0.97)	na	-0.03 (0.83)	na	0.11 (0.80)	na	-0.00 (1.00)	na	0.15 (0.34)	na
AC term	0.29 (0.08)	0.14 (0.41)	0.52 (**<0.001**)	0.33 (**0.04**)	-0.29 (0.46)	0.48 (0.07)	-0.01 (0.75)	0.03 (0.38)	0.30 (0.11)	0.65 (**<0.001**)

	Medium mammal richness		Large mammal richness		Primate richness		Ungulate richness		Human	
Model	Full	Null	Full	Null	Full	Null	Full	Null	Full	Null
AIC	327.2	329.4	313.4	311.7	292.7	310.2	296.2	285.5	631.2	748.7
No. parameter	8	2	8	2	8	2	8	2	8	2
Intercept	0.54 (**<0.001**)	0.56 (**<0.001**)	0.13 (**0.05**)	0.14 (**0.02**)	-0.26 (**<0.001**)	-0.18 (**0.01**)	0.18 (**<0.01**)	0.18 (**<0.01**)	1.28 (**<0.001**)	1.36 (<**0.001**)
Slope	0.01 (0.84)	na	0.07 (0.31)	na	0.15 (**0.05**)	na	-0.01 (0.88)	na	-0.04 (0.26)	na
Road	0.08 (0.13)	na	0.06 (0.33)	na	0.11 (0.13)	na	0.04 (0.52)	na	-0.32 (<**0.001**)	na
Primary forest	-0.10 (0.07)	na	0.01 (0.90)	na	-0.03 (0.72)	na	-0.02 (0.77)	na	0.02 (0.45)	na
Settlement	0.16 (**0.02**)	na	0.08 (0.32)	na	0.28 (**<0.01**)	na	0.04 (0.59)	na	-0.10 (**0.01**)	na
City	-0.03 (0.59)	na	0.05 (0.41)	na	-0.02 (0.80)	na	0.02 (0.81)	na	-0.17 (<**0.001**)	na
PA	-0.02 (0.68)	na	0.04 (0.44)	na	-0.02 (0.79)	na	-0.02 (0.80)	na	0.07 (0.07)	na
AC term	-0.15 (<**0.01**)	-0.10 (**0.05**)	0.22 (**<0.01**)	0.28 (<**0.001**)	0.11 (0.20)	0.15 (0.05)	-0.06 (0.32)	-0.05 (0.40)	0.30 (<**0.001**)	0.34 (<**0.001**)

Coefficient values are followed by the *p*-value in brackets. Significant *p*-values are displayed in bold.

Nest decay rates were used to give a population estimate of 9,232 (6,059–14,064) apes in Distance and 11,097 (5,090–20,688) apes according to the spatial model in Río Muni. A separate model for gorillas could not be generated as gorilla nests were only recorded along five of the transects totalling just 51 nests.

Proximity to cities and settlements were the strongest predictors of ape density, which decreased closer to these human centres (Figure 2; Table 4, Table 5). Autocorrelation was highly significant but forest, slope, distance to roads and PA were not good predictors (Table 4, Table 5).

In the second model, in which agricultural mosaic habitat was substituted for roads, both chimpanzees and apes collectively showed a significant negative correlation with this habitat type (Figure S2; Table S5).

### Elephants

During the line transect surveys, 199 elephant dung piles were encountered along 21 of the transects. The design-based Distance estimate was 884 (437–1,789) elephants.

Slope and proximity to roads, cities and settlements were the important predictors for elephants. Elephant abundance increased with increasing distance away from roads, cities and settlements (Figure 2). Slope revealed a positive effect indicating that elephants are more abundant on steeper slopes. Other variables were found not to be significant.

### Mammals

Increasing distance away from roads and settlements revealed a significant increase in mammal species richness but none of the other covariates were good predictors.

Body mass of all mammals combined showed a negative correlation with increasing proximity to settlements but was not significantly affected by any of the other covariates. When separated into large and medium mammals according to body mass, the former revealed only a significant negative relationship with agricultural mosaic habitat out of the two models (Table 4, Table 5 & Table S4). For medium-bodied mammals, proximity to settlements and agricultural mosaic habitat illustrated a negative impact on population.

Primate richness was determined very strongly by the distance to settlements and less strongly by slope. The second model showed a significant negative correlation between primate richness and agricultural mosaic habitat. Ungulate richness appeared not to be affected by any of the covariates tested.

### Humans

Human signs along the transects were clearly more abundant closer to roads, settlements and cities and thus showed the expected opposite pattern of wildlife distribution.

## Discussion

The nationwide population size estimate of apes in EG in 2011 stands at 11,097, which consists of a maximum population of 7,824 chimpanzees and a minimum population of 3,273 gorillas. A precise estimation could not be made due to the possible misclassification of the two ape species’ nests and due to the small sample size of gorilla nests. We estimate an elephant population of almost 900 individuals, most of which are present within PNMA. Human signs were high and were encountered frequently along the majority of transects surveyed. Apes, elephants and large mammals in general were most abundant within PNMA and the region extending south towards Gabon, which is part of the Monte Alén-Monts de Cristal transboundary landscape (MAMC). A second notably important area is the Río Campo Nature Reserve (RNRC) and the region to the east of the reserve. Using these findings, we propose these as priority areas for the conservation of the remaining large mammal populations on Río Muni.

### Differences in Design and Model based Abundance Estimates

Comparing the design-based abundance estimate for apes (9,232) with the one derived from spatial model prediction (11,097) suggests that our systematic design with transect spacing of 18 km may not have been sufficient to capture countrywide mean ape density as observed on transect locations in an unbiased way: a greater difference was seen in the derived population size estimate than the density estimate (Text S3; Figure S3). This suggests that ape density observed on transects is likely an underestimate of countrywide density. However, each estimate is well within the confidence limits of the respective other method.

### Commercial vs. Local Hunting

The significant effects of subsistence and commercial bushmeat hunting are evident from the results. The abundance of apes and elephants was clearly determined by the distance to cities, and for almost all response variables tested, negative correlation with increasing proximity to settlements were shown. The only response variables that did not show any significance with distance to settlements were the large mammals and ungulates.

For small-bodied mammals such as rodents and blue duiker (*Philantomba monticola*), there is a general understanding on their strong resilience to survive in human-dominated landscapes. Our finding, that the abundance of ungulates is not significantly affected by any of the predictor variables, supports the post-depletion hypothesis [16], whereby only the fast reproducing species remain due to preferential hunting of larger mammals as a result of cost-benefit trade-offs [63], [64]. The apparent lack of significant impact on large mammal richness in this study would therefore appear to be contradictory to what is expected according to this theory. However, this can be explained on closer inspection of the transect data, where the extremely low encounter rate of large mammal signs becomes evident. The finding can therefore be a reflection of the already low population numbers and fragmented range of the large-bodied mammals across Río Muni.

Although proximity to cities only served statistically as a strong indicator for chimpanzees, apes and elephants, the significant prevalence of commercial hunting on other species should not be underestimated. Extensive commercial hunting in EG and the presence of large urban bushmeat markets in Río Muni are well known and have been exclusively studied [11], [65]. Bushmeat is even supplied to major cities from some of the less-accessible catchments [11], suggesting great urban demand. An explanation for the distance to cities not appearing significant for some of the response variables could be due to the extensive tarmac road network that now exists throughout the country. Most settlements are now connected by a good road to a major city and hence distance to cities has become less of an issue in transportation in recent years.

Intriguingly, only elephant and overall mammal richness showed a significant relationship with distance to roads. This signifies that bushmeat hunters are no longer reliant on accessibility or distance to roads, perhaps due to the already extensive hunting and decline of wildlife throughout the country. This would support the findings that hunters in Río Muni now have to travel further to hunt, since wildlife populations have been depleted in the vicinity of most towns, villages and roads [15]. Furthermore, this would indicate that the extent of wildlife decline in Río Muni surpasses the situation in surrounding Central African countries, where road proximity still acts as the most significant impact on poaching [12], [13], [66], [67].

A comparison of our results with previous studies seems logical in this context, however, due to differences in methodology and spatial scales, such analysis is not straightforward (Text S4).

### Effectiveness of PAs

Worryingly, PAs had no statistical significance on large mammal distribution in Río Muni, illustrating the lack of law enforcement, patrols and protection. Although the existence of ‘paper parks’ is widely understood [23], there are many examples where PAs do offer adequate protection and make significant differences on mammal conservation when proper management is reinforced [12], [18], [19], [68], [69]. The shortage of operating funds that INDEFOR-AP in EG has experienced has hindered the deployment of guards, resulting in limited or no law enforcement or control within PAs.

**Figure 3:**
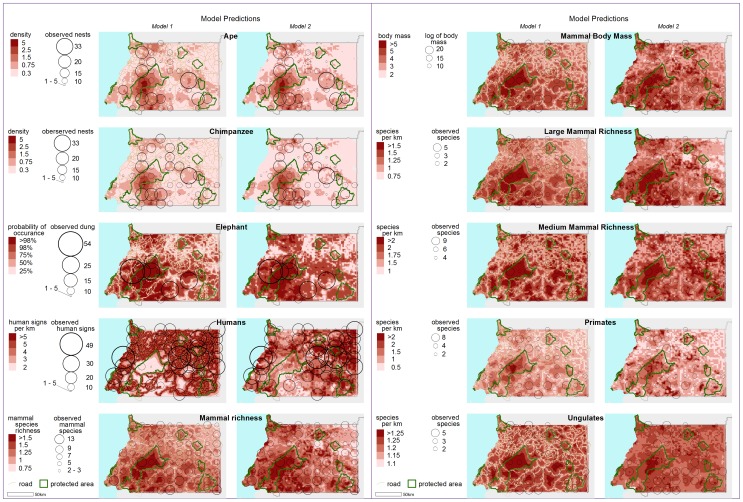
Nationwide distribution maps. Model 1 includes the variable for distance roads. Model 2 includes agricultural mosaic habitat instead of distance to roads. Number of encounter signs along the line transects are represented proportionally as black open circles.

Despite this finding, there does appear to be a concentration of elephants, apes and mammal body mass within the PNMA (Figure 3). This apparent hotspot could be explained by the topographical nature of the Park. The steep slopes of the Niefang mountain range contained within PNMA, combined with the remoteness of the site, could be the contributing factor for creating this refuge for large mammals. In particular, steeper slopes were found to be statistically significant in determining elephant and primate presence. Furthermore, Miserga, the closest village to the Park on the eastern side, was once a sizeable community but the younger generations have left for the cities in recent years, and this may have eased the pressure on wildlife in this region [M.M. personal observation]. Though the migration of the younger generations out of villages is a general trend seen across the country, the difficult road conditions to access Miserga appear to be acting as a deterrent for external commercial hunters.

The hotspot was not exclusively limited to within the PNMA, but also in the area extending south towards Monts de Cristal National Park in Gabon (Figure 3). MAMC was identified as a priority site for the Central African Regional Program for the Environment (CARPE) of the United States Agency for International Development (USAID) due to the botanical importance and diversity as a result of past Pleistocene refugia [29]. It is now possible to add from this study that focus can be placed not only on the vegetation, but also on the mammal species that the landscape holds. MAMC encompasses an area of 26,747 km^2^ which includes PNMA, Altos de Nsork NP, the Estuary of Muni Nature Reserve and Piedras Nzas Natural Monument (MNPN) and the proposed National Forest (BNGE) on the EG side [29]. In order to implement protection most efficiently, we suggest that conservation activities be focused on the western half of the landscape on the EG side in order to prioritise the available resources. The development plan to transform Oyala [70], close to MNPN and Piedra Bere Natural Monument, into a new capital city is well underway, and, given the rapid rate of infrastructural development, efforts on the eastern half of the landscape should be focused on minimising damages to the PAs.

In addition to the importance of the PNMA region, this study appears to show another important, though much smaller area, in RNRC and the region just to the east of the reserve. It is difficult to determine if this is indeed the case, as the transect in that region fell outside of the country and was not surveyed as a result, and we hence cannot rule out the extent of modelling error. On the other side of the border lies the Campo Ma’an National Park (CMNP) in Cameroon, however, which extends down to Río Muni. Although the gorilla population is low in CMNP, the chimpanzee density was found to be high compared to other parts of the country and the rest of West and Central Africa in earlier studies [71]–[73]. Furthermore, it is understood that the current elephant population to the east of RNRC crossed over from CMNP [12]. Although a transboundary complex, the ‘Río Campo Ma’an’ initiative, has been launched, shortage of funding has prevented further developments beyond its initial stages. Financial security is a priority and there is additionally the need to expand the Río Campo Ma’an complex to include the regions to the east of RNRC.

### Ecological Factors vs. Human Impact

Overall, human disturbance was found to override the importance of ecological factors. Distance to cities and especially settlements appeared to be the most important predictors for most mammals, illustrating the strong influence of bushmeat hunting in Río Muni. Commercial hunting has been exacerbated by the ease in the firearm ban in 1979, meaning both guns and cartridges are now more affordable and accessible. Though snare traps may still be the dominant form of hunting, the large number of hunters we observed with guns may be an indicator of the sufficient profit they make through urban markets, as the 500 FCFA (ca.1 USD) price tag per standard cartridge is equivalent to the average daily household income in EG [65].

Human disturbance, however, is not only caused by bushmeat hunting but also occurs as a result of human wildlife conflict. The elephant population in RNRC was demolished in the mid-1980s when crop-raiding by elephants led to the government’s action of eliminating the elephant population [33]. Crop raiding has been identified as an increasing problem in many villages, in particular around PNMA in a recent study [74], and is leading to severe conflicts, solved by the government through ‘administrative killing’ [75]. On the policy level, elephants are in fact relatively well protected in EG, legally requiring an official request in order to cull an elephant due of human elephant conflict (HEC) [74]. Once the request is accepted, a government-approved elephant hunter is allowed to cull a single individual, whose tusks are subsequently given to the government [74]. In practice, however, culling is not controlled and one of several elephant hunters who we encountered during the fieldwork admitted having already killed five in the past week [M.M. personal observation]. The extent to which this is happening in the country is impossible to judge from the current study, and the degree to which personal communications should be believed needs to be taken with caution, but it still highlights the lack of wildlife protection in the country. This is a striking observation that must be dealt with immediately to implement control of the elephant killing, and come up with an alternative method to mitigate HEC, especially given the lack of livelihood alternatives in the villages. Greater emphasis should be made on transboundary cooperation on conservation to accommodate for the migrating elephants and other mammal species.

Agricultural mosaic habitat was a strong negative predictor for large mammal richness, primate richness and ape abundance. Chimpanzees are frequently associated with primary and old secondary forests [50], [73], [76], [77] so our study is consistent with past studies, but is contrary to the finding that chimpanzees are also documented to use habitats in human settlement proximity and human-modified niches [78]. There may be site- or country-specific differences affected by several factors such as spatial and temporal availability of food resources and hunting pressure that are contributing to this phenomenon.

### Conclusion and Recommendations

Our findings on the detrimental impact of hunting and the negative impact of croplands in Río Muni are extremely concerning given the rapid infrastructural development throughout the country. With the economic development still underway, we can expect further devastating impacts on wildlife, and this study allows us to take a part-way snapshot of the effect it is having. Furthermore we were able to show the feasibility of a nationwide survey using this methodology in a relatively small country which can be amplified and applied to other larger countries.

Conservation efforts need to be more focused in areas containing the remaining wildlife populations, implying that the boundaries of transboundary conservation landscapes or at least the priority activities within these should be revised when updated data are available. Some regions outside of PNMA and RNRC contain ape and elephant populations that are higher in density than inside the PAs. Both of these PAs hence need to be expanded to give greater protection to these remaining populations. Most importantly, law enforcement needs to be implemented for PA management across EG. We have shown the effect of ‘paper parks’ in Río Muni and have seen in other countries that with sufficient enforcement, PAs can have a significantly positive impact on mammal conservation. We strongly recommend the increased deployment of guards and an uninterrupted supply of operating funds for PA management.

### Public Access to Data

All raw data from the survey on apes are archived into the IUCN/SSC A.P.E.S. database (http://apes.eva.mpg.de/) [79]. Elephant data will be uploaded into the IUCN/SSC African Elephant Specialist Group’s database (http://www.elephantdatabase.org). These data will be accessible by third parties on request.

## Supporting Information

Figure S1 **Histograms of detection distances and detection functions.** Top: Chimpanzee, Middle: Apes, Bottom: Elephants.
(DOC)

Figure S2 **Plots of covariate effect for model 2.** Observations (circles) and model predictions (lines) are shown for every combination of response and predictor. Transects are grouped and circle sizes are proportional to the number of transects. Bold lines represent significant effects. Cropland = agricultural mosaic habitat; forest = primary forest.
(DOC)

Figure S3 **Comparison of ape abundance estimate derived from spatial model predictions and design-based inference.**
(DOC)

Table S1 **Total number of signs and the observation types recorded per species across 83 transects and their body mass.**
(DOC)

Table S2 **Predictor variables initially selected.**
(DOC)

Table S3 **Spearman’s correlation values for model 1 and model 2.**
(DOC)

Table S4 **Settings and results from Distance analysis.**
(DOC)

Table S5 **Full and null model results for the second model.**
(DOC)

Text S1 **Distinguishing chimpanzee and gorilla nests.**
(DOC)

Text S2 **Autocorrelation.**
(DOC)

Text S3 **Comparison of design based and spatial model predictions.**
(DOC)

Text S4 **Comparison with previous estimates.**
(DOC)
