# Supplementary material for: Priority Areas for Large Mammal Conservation in Equatorial Guinea
Source: PLoS One. 2013 Sep 27;8(9):e75024. doi: 10.1371/journal.pone.0075024 (PMC3785506; doi:10.1371/journal.pone.0075024)
Supplement: Figure S1 — Histograms of detection distances and detection functions. Top: Chimpanzee, Middle: Apes, Bottom: Elephants. (DOC) [file pone.0075024.s001.doc]

**
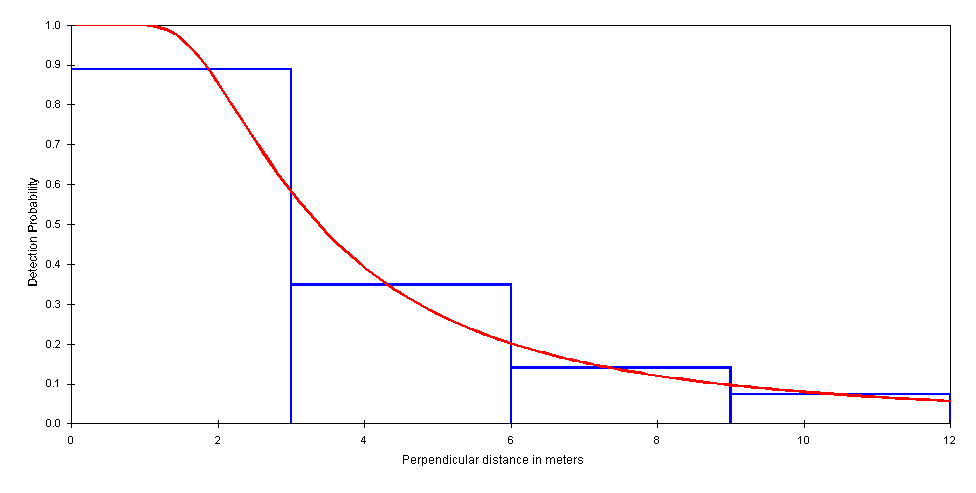

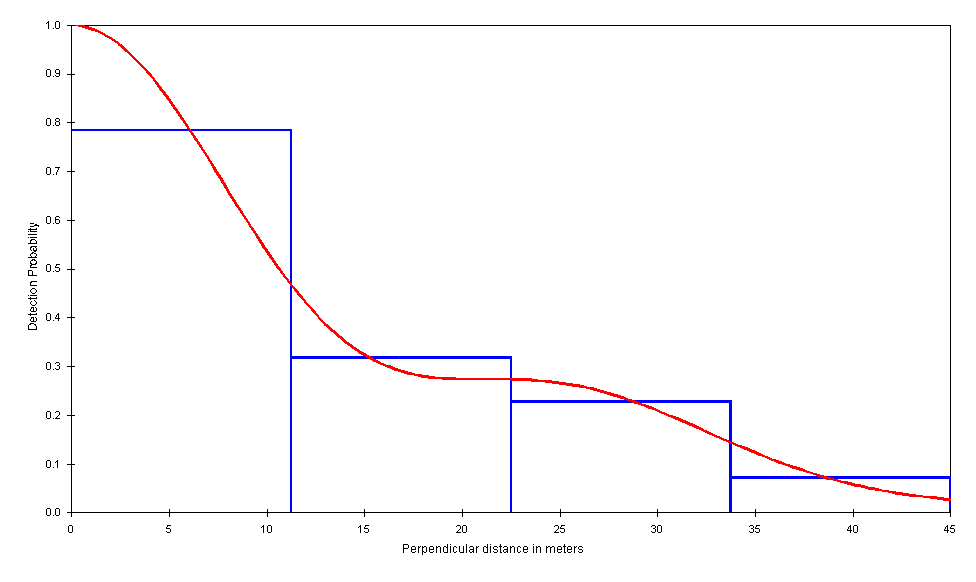

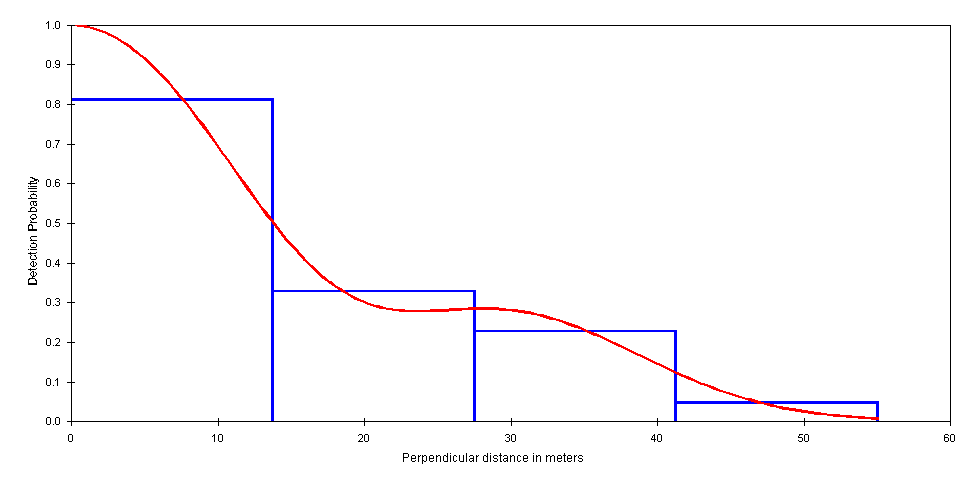
**

**Figure S1. Histograms of detection distances and detection functions.** Top: Chimpanzee, Middle: Apes, Bottom: Elephants.
