# Supplementary material for: Priority Areas for Large Mammal Conservation in Equatorial Guinea
Source: PLoS One. 2013 Sep 27;8(9):e75024. doi: 10.1371/journal.pone.0075024 (PMC3785506; doi:10.1371/journal.pone.0075024)
Supplement: Figure S2 — Plots of covariate effect for model 2. Observations (circles) and model predictions (lines) are shown for every combination of response and predictor. Transects are grouped and circle sizes are proportional to the number of transects. Bold lines represent significant effects. Cropland = agricultural mosaic habitat; forest = primary forest. (DOC) [file pone.0075024.s002.doc]

**
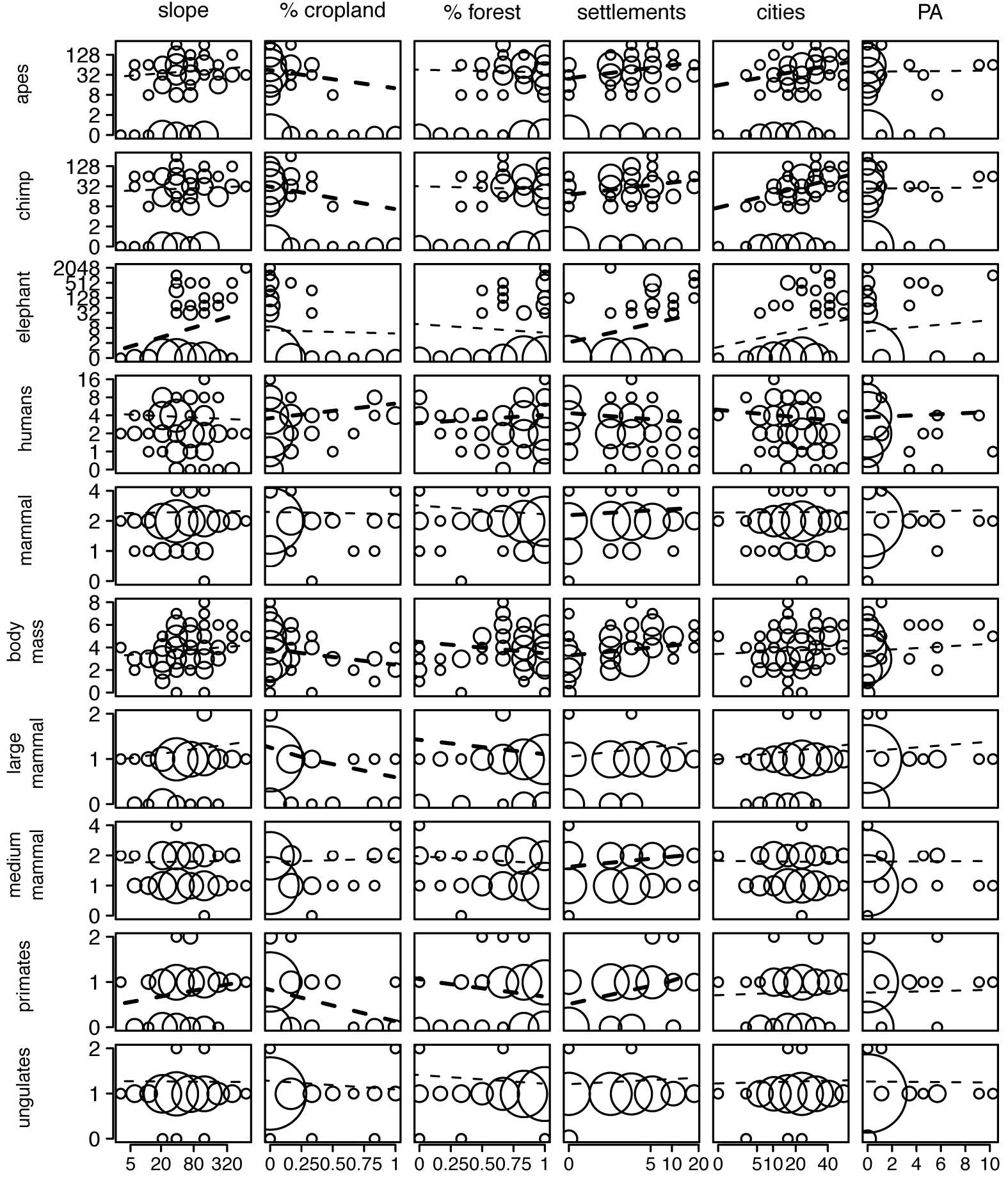
**

**Figure S2. Plots of covariate effect for model 2.** Observations (circles) and model predictions (lines) are shown for every combination of response and predictor. Transects are grouped and circle sizes are proportional to the number of transects. Bold lines represent significant effects. Cropland = agricultural mosaic habitat; forest = primary forest**.**
