# Supplementary material for: Priority Areas for Large Mammal Conservation in Equatorial Guinea
Source: PLoS One. 2013 Sep 27;8(9):e75024. doi: 10.1371/journal.pone.0075024 (PMC3785506; doi:10.1371/journal.pone.0075024)
Supplement: Figure S3 — Comparison of ape abundance estimate derived from spatial model predictions and design-based inference. (DOC) [file pone.0075024.s003.doc]

**Figure S3. Comparison of ape abundance estimate derived from spatial model predictions and design-based inference.**
