# Supplementary material for: Priority Areas for Large Mammal Conservation in Equatorial Guinea
Source: PLoS One. 2013 Sep 27;8(9):e75024. doi: 10.1371/journal.pone.0075024 (PMC3785506; doi:10.1371/journal.pone.0075024)
Supplement: Table S1 — Total number of signs and the observation types recorded per species across 83 transects and their body mass. (DOC) [file pone.0075024.s004.doc]

**Table S1. Total number of signs and the observation types** recorded per species across 83 transects and their body mass.

| Genus | Species | English common names | Different mammal signs recorded | | | | | | | Total no. of signs | Body mass, kg [source] |
| --- | --- | --- | --- | --- | --- | --- | --- | --- | --- | --- | --- |
|  |  |  | Direct sighting | Faeces | Feeding remains | Nest | Tools | Tracks/ prints | Vocalisation |  |  |
| *Aonyx* | *congicus* | Congo clawless otter | - | - | - | - | - | 12 | - | 12 | 20.0 [35] |
| *Atherurus* | *africanus* | African bush-tailed porcupine | 5 | 8 | 9 | - | - | 320 | - | 342 | 2.8 [36] |
| *Cephalophus* | *dorsalis* | Bay duiker* | 15 | 101 | 1 | - | - | 326 | - | 443 | 20.4 [36] |
| *Cephalophus* | *silvicultor* | Yellow-backed duiker* | - | 2 | - | - | - | 10 | - | 12 | 52.5 [36] |
| *Cercopithecus* | *cephus* | Moustached monkey^†^ | 4 | - | 3 | - | - | - | 3 | 10 | 3.5 [36] |
| *Cercopithecus* | *nictitans* | Putty-nosed monkey^†^ | 8 | - | 1 | - | - | - | 31 | 40 | 5.0 [36] |
| *Cercopithecus* | *pogonias* | Crowned monkey^†^ | 3 | - | - | - | - | - | 16 | 19 | 3.8 [36] |
| *Civettictis* | *civetta* | African civet | 1 | 4 | - | - | - | 4 | - | 9 | 12.4 [36] |
| *Colobus* | *satanas* | Black Colobus^†^ | 2 | - | - | - | - | - | 5 | 7 | 12.5 [36] |
| *Crossarchus* | *platycephalus* | Cameroon cusimanse | - | - | - | - | - | 7 | - | 7 | 1.3 [37] |
| *Cusimanse* | unidentified | Cusimanse | - | - | - | - | - | 2 | - | 2 | 1.3 [37] |
| *Funisciurus* | *isabella* | Lady Burton’s rope squirrel | 1 | - | - | - | - | - | - | 1 | 0.2 [35] |
| *Euoticus* | *elegantulus* | South needle-clawed galago^†^ | 1 | - | - | - | - | - | - | 1 | 0.3 [36] |
| *Gorilla* | *g. gorilla* | Western lowland gorilla^†^ | - | 9 | 22 | 53 | - | 13 | 1 | 98 | 133.0 [36] |
| *Herpestes* | *naso* | Long-nosed mongoose | - | - | 1 | - | - | 9 | - | 10 | 3.6 [35] |
| *Hyemoschus* | *aquaticus* | Water chevrotain* | - | 3 | - | - | - | 1 | - | 4 | 15.0 [36] |
| *Loxodonta* | *cyclotis* | African forest elephant | - | 199 | 4 | - | - | 224 | - | 427 | 2575.0 [36] |
| *Mandrillus* | *sphinx* | Mandrill^†^ | - | 3 | 40 | - | - | 13 | 1 | 57 | 17.4 [36] |
| *Miopithecus* | *ogouensis* | Northern talapoin monkey^†^ | 2 | - | - | - | - | 1 | - | 3 | 1.3 [35] |
| *Myosciurus* | *pumilio* | African pygmy squirrel | 1 | - | - | 2 | - | - | - | 3 | 0.2 [35] |
| *Nandinia* | *binotata* | African palm civet | - | - | - | - | - | 1 | - | 1 | 3.0 [36] |
| *Neotragus* | *batesi* | Bates’ pygmy antelope* | - | - | 2 | - | - | - | - | 2 | 2.7 [36] |
| *Pan* | *t. troglodytes* | Central chimpanzee^†^ | - | 5 | 8 | 332 | 3 | 10 | 2 | 360 | 45.0 [36] |
| *Panthera* | *pardus* | Leopard | - | - | - | - | - | 5 | - | 5 | 47.5 [36] |
| *Philantomba* | *monticola* | Blue duiker* | 13 | 70 | - | - | - | 178 | - | 261 | 4.9 [36] |
| *Potamochoerus* | *porcus* | Red river hog* | - | 4 | 3 | 1 | - | 243 | 1 | 252 | 67.5 [36] |
| *Protoxerus* | *stangeri* | African giant squirrel | 2 | - | - | - | - | - | - | 2 | 0.8 [35] |
| *Smutsia* | *gigantea* | Giant ground pangolin | - | - | 5 | - | - | 118 | - | 123 | 32.5 [35] |
| *Syncerus* | *caffer* | African buffalo* | - | - | - | - | - | 6 | - | 6 | 285.0 [36] |
| *Thryonomys* | *swinderianus* | Greater cane rat | 1 | - | 3 | - | - | 30 | - | 34 | 5.1 [35] |
| *Tragelaphus* | *spekeii* | Sitatunga* | 1 | 7 | 1 | - | - | 111 | - | 120 | 100.0 [36] |
| *Uromanis* | *tetradactyla* | Black-bellied pangolin | - | - | 2 | - | - | 42 | - | 44 | 2.3 [35] |

*Species that were included in the ungulate analyses; ^†^Species that were included in the primate analyses.
