# Supplementary material for: Priority Areas for Large Mammal Conservation in Equatorial Guinea
Source: PLoS One. 2013 Sep 27;8(9):e75024. doi: 10.1371/journal.pone.0075024 (PMC3785506; doi:10.1371/journal.pone.0075024)
Supplement: Table S2 — Predictor variables initially selected. (DOC) [file pone.0075024.s005.doc]

**Table S2**. **Predictor variables initially selected**

| **Variable** | **Resolution** | **Unit** | **Description** | **Source** | **Website** |
| --- | --- | --- | --- | --- | --- |
| Altitude | 1000 | Metre |  | CGIAR SRTM | http://srtm.csi.cgiar.org/ |
| Slope | 1000 | Metre | Mean slope | CARPE | http://congo.iluci.org:8080/geonetwork/srv/en/main.home |
| River | 632 | Metre | Euclidean distance to rivers | INDEFOR-AP | na |
| Land cover | 311 | Metre | Frequency of class ht_30 and ht_40_160 from the centre of the transect within a diameter half the length of the transect | ESA 2009 | http://spaceinimages.esa.int/Images/2010/12/ESA_s_2009_global_land_cover_map |
| Soil | 5 | Arc-min | Dominant soil groups | FAO 2000 | http://www.fao.org/nr/land/suelos/soil/wrb-soil-maps/wrb-map-of-world-soil-resources/es/ |
| Cities | 1000 | Metre | Euclidean distance to cities | INDEFOR-AP | na |
| Settlements | 1000 | Metre | Euclidean distance to settlements | INDEFOR-AP | na |
| Roads | 1000 | Metre | Euclidean distance to roads | WRI | na |
| PA | 1000 | Metre | Binary categorisation of PA or non-PA | CARPE | http://congo.iluci.org:8080/geonetwork/srv/en/main.home |
| Distance inside PA | 1000 | Metre | Euclidean distance within a PA | CARPE | na |
| Poverty |  |  |  | World Bank | http://data.worldbank.org/topic/poverty |
| Precipitation | 10 | Arc-min |  | WorldClim | http://www.worldclim.org/current |
| Mean temperature | 10 | Arc-min |  | WorldClim | http://www.worldclim.org/current |
| Temperature range | 10 | Arc-min |  | WorldClim | http://www.worldclim.org/current |
