# Supplementary material for: Priority Areas for Large Mammal Conservation in Equatorial Guinea
Source: PLoS One. 2013 Sep 27;8(9):e75024. doi: 10.1371/journal.pone.0075024 (PMC3785506; doi:10.1371/journal.pone.0075024)
Supplement: Table S3 — Spearman’s correlation values for model 1 and model 2. (DOC) [file pone.0075024.s006.doc]

**Table S3. Spearman’s correlation values for model 1 and model 2.**

|  | **Predictor variables** | **Slope** | **Road / Agricultural mosaic** | **Primary forest** | **Settlement** | **City** | **PA** |
| --- | --- | --- | --- | --- | --- | --- | --- |
| Model 1 | Slope | 1 | 0.20 | 0.27 | 0.12 | 0.32 | 0.18 |
|  | Road | 0.20 | 1 | 0.29 | 0.47 | 0.46 | 0.09 |
|  | Primary forest | 0.27 | 0.29 | 1 | 0.18 | 0.19 | 0.06 |
|  | Settlement | 0.12 | 0.47 | 0.18 | 1 | 0.29 | 0.19 |
|  | City | 0.32 | 0.46 | 0.19 | 0.29 | 1 | 0.10 |
|  | PA | 0.18 | 0.09 | 0.06 | 0.19 | 1.10 | 1 |
| Model 2 | Slope | 1 | -0.42 | 0.27 | 0.12 | 0.32 | 0.18 |
|  | Agricultural mosaic | -0.42 | 1 | -0.77 | -0.21 | -0.27 | -0.25 |
|  | Primary forest | 0.27 | -0.77 | 1 | 0.18 | 0.19 | 0.06 |
|  | Settlement | 0.12 | -0.21 | 0.18 | 1 | 0.29 | 0.19 |
|  | City | 0.32 | -0.27 | 0.19 | 0.29 | 1 | 0.10 |
|  | PA | 0.18 | -0.25 | 0.06 | 0.19 | 0.10 | 1 |

Model 1 included distance to roads as a variable; Model 2 replaced distance to roads with agricultural mosaic habitat.
