# Supplementary material for: Priority Areas for Large Mammal Conservation in Equatorial Guinea
Source: PLoS One. 2013 Sep 27;8(9):e75024. doi: 10.1371/journal.pone.0075024 (PMC3785506; doi:10.1371/journal.pone.0075024)
Supplement: Table S4 — Settings and results from Distance analysis. (DOC) [file pone.0075024.s007.doc]

**Table S4. Settings and results from Distance analysis.**

| **Species** | **Truncation distance** | **ESW** | **No. parameter** | **Key** | **Adjustment** | **AIC** | **N** | **N LCL** | **N UCL** |
| --- | --- | --- | --- | --- | --- | --- | --- | --- | --- |
| Chimpanzee | 55 | 19.49 | 3 | Half-normal | Simple polynomials | 695.53 | 6,225 | 4,066 | 9,530 |
| Chimpanzee | 55 | 18.35 | 3 | Half-normal | Cosines | 695.53 | 6,610 | 4,279 | 10,211 |
| Apes | 45 | 15.77 | 3 | Half-normal | Cosines | 826.66 | 9,232 | 6,059 | 14,064 |
| Elephants | 12 | 0.0436 | 2 | Half-normal | Cosines | 404.17 | 910 | 456 | 1,814 |
| Elephants | 12 | 0.041 | 2 | Hazard rate | Hermite polynomials | 404.17 | 857 | 417 | 1,763 |

For both chimpanzees and elephants two models had the same lowest AIC of all models evaluated. Abundance estimates were therefore averaged. Chimpanzees: 6,418 (4,173-9,871); Elephants: 884 (437-1,789). ESW=effective strip width; N=abundance estimate; N LCL=lower confidence limit; N UCL= upper confidence limit.
