# Supplementary material for: Priority Areas for Large Mammal Conservation in Equatorial Guinea
Source: PLoS One. 2013 Sep 27;8(9):e75024. doi: 10.1371/journal.pone.0075024 (PMC3785506; doi:10.1371/journal.pone.0075024)
Supplement: Table S5 — Full and null model results for the second model. (DOC) [file pone.0075024.s008.doc]

**Table S5. Full and null model results for the second model.**

|  | **Chimpanzee** | |  | **Ape** | |  | **Elephant** | |  | **All mammal richness** | |  | **Mammal body mass** | |
| --- | --- | --- | --- | --- | --- | --- | --- | --- | --- | --- | --- | --- | --- | --- |
| **Model** | **Full** | **Null** |  | **Full** | **Null** |  | **Full** | **Null** |  | **Full** | **Null** |  | **Full** | **Null** |
| AIC | 377.1 | 402.9 |  | 402.5 | 423.1 |  | 63.3 | 82.0 |  | 394.3 | 403.5 |  | 294.1 | 306.4 |
| No. parameter | 8 | 2 |  | 8 | 2 |  | 8 | 2 |  | 8 | 2 |  | 8 | 2 |
| Intercept | 3.10 (<**0.001**) | 3.59 (<**0.001**) |  | 3.52 (<**0.001**) | 3.91 (<**0.001**) |  | 1.30  (**0.03**) | 2.31 (<**0.001**) |  | 1.06  (<**0.001**) | 1.08  (<**0.001**) |  | 3.73 (<**0.001**) | 3.73 (<**0.001**) |
| Slope | 0.02  (0.92) | na |  | 0.21  (0.16) | na |  | 1.60  (**0.01**) | na |  | 0.05  (0.22) | na |  | 0.29  (0.06) | na |
| Agricultural mosaic | -1.09 (<**0.01**) | na |  | -0.83  (**0.03**) | na |  | -0.24  (0.81) | na |  | -0.19  (0.05) | na |  | -1.22  (**<0.01**) | na |
| Primary forest | -0.67  (0.06) | na |  | -0.57  (0.11) | na |  | -1.15  (0.28) | na |  | -0.22  (0.02) | na |  | -1.17  (<**0.01**) | na |
| Settlement | 0.52  (<**0.01**) | na |  | 0.57 (<**0.001**) | na |  | 1.62  (<**0.01**) | na |  | 0.16  (**<0.001**) | na |  | 0.55  (<**0.01**) | na |
| City | 0.74 (<**0.001**) | na |  | 0.49  (<**0.01**) | na |  | 0.86  (0.09) | na |  | 0.00  (0.97) | na |  | -0.08  (0.62) | na |
| PA | 0.01  (0.95) | na |  | -0.00  (0.99) | na |  | 0.25  (0.50) | na |  | 0.01  (0.89) | na |  | 0.14  (0.35) | na |
| AC term | 0.24  (0.14) | 0.10  (0.55) |  | 0.46  (<**0.01**) | 0.29  (0.08) |  | 0.60  (0.16) | 0.86 (<**0.01**) |  | -0.01  (0.76) | 0.04  (0.36) |  | 0.30  (0.07) | 0.55  (**<0.01**) |

|  | **Medium mammal richness** | |  | **Large mammal richness** | |  | **Primate richness** | |  | **Ungulate richness** | |  | **Human** | |
| --- | --- | --- | --- | --- | --- | --- | --- | --- | --- | --- | --- | --- | --- | --- |
| **Model** | **Full** | **Null** |  | **Full** | **Null** |  | **Full** | **Null** |  | **Full** | **Null** |  | **Full** | **Null** |
| AIC | 330.8 | 329.6 |  | 307.22 | 315.3 |  | 285.7 | 311.7 |  | 295.9 | 285.5 |  | 644.1 | 742.0 |
| No. parameter | 8 | 2 |  | 8 | 2 |  | 8 | 2 |  | 8 | 2 |  | 8 | 2 |
| Intercept | 0.54  (**<0.001**) | 0.56  (**<0.001**) |  | 0.11  (0.09) | 0.15  (**0.01**) |  | -0.28  (**<0.001**) | -0.17  (**0.02**) |  | 0.18  (**<0.01**) | 0.18  (**<0.01**) |  | 1.3 (**<0.001**) | 1.35 (<**0.001**) |
| Slope | 0.02  (0.76) | na |  | 0.08  (0.23) | na |  | 0.17  (**0.04**) | na |  | -0.01  (0.92) | na |  | -0.07 (0.06) | na |
| Agricultural mosaic | 0.01  (0.93) | na |  | -0.45  (**<0.01**) | na |  | -0.60  (**<0.01**) | na |  | -0.14  (0.37) | na |  | 0.49 (<**0.001**) | na |
| Primary forest | -0.10  (0.43) | na |  | -0.33  (0.02) | na |  | -0.52  (**<0.01**) | na |  | -0.14  (0.35) | na |  | 0.42 (<**0.001**) | na |
| Settlement | 0.17  (**<0.01**) | na |  | 0.11  (0.14) | na |  | 0.35  (**<0.001**) | na |  | 0.06  (0.41) | na |  | -0.18 (<**0.001**) | na |
| City | -0.03  (0.59) | na |  | 0.05  (0.43) | na |  | -0.04  (0.63) | na |  | 0.01  (0.86) | na |  | -0.13 (<**0.001**) | na |
| PA | -0.01  (0.86) | na |  | 0.03  (0.58) | na |  | -0.03  (0.68) | na |  | -0.02  (0.81) | na |  | 0.12 (<**0.01**) | na |
| AC term | -0.13  (**0.01**) | -0.09  (0.06) |  | 0.19  (**<0.01**) | 0.23  (<**0.001**) |  | 0.03  (0.72) | 0.11  (0.12) |  | -0.06  (0.33) | -0.05  (0.42) |  | 0.38 (<**0.001**) | 0.36 (<**0.001**) |

Coefficient values are followed by the *p*-value in brackets. Significant *p*-values are displayed in bold.
