# Supplementary material for: Priority Areas for Large Mammal Conservation in Equatorial Guinea
Source: PLoS One. 2013 Sep 27;8(9):e75024. doi: 10.1371/journal.pone.0075024 (PMC3785506; doi:10.1371/journal.pone.0075024)
Supplement: Text S1 — Distinguishing chimpanzee and gorilla nests. (DOC) [file pone.0075024.s009.doc]

**Text S1.** **Distinguishing chimpanzee and gorilla nests**

Reliably distinguishing between chimpanzee and gorilla nests only by visual inspection is challenging [43]. Though the two species construct both ground and arboreal nests, characteristics such as a group of large ground nests can be attributed to gorillas, whereas a group of smaller tree nests may be identified as belonging to chimpanzees. It is possible however, that some of the tree nests in our study were misidentified. On one occasion, a nest was found 20 m up a tree which was able to be identified as a gorilla’s due to direct observations by villagers of a gorilla in that particular nest a couple of days ago prior to our observation. As only one nest was found in that area, providing no further reference points, it is likely that this nest would have been categorised as a chimpanzee’s had we not had this information.

Based on these observations, we can assume that the derived ‘chimpanzee’ abundance estimate of 7,824 individuals also contains an unknown number of gorillas, as some of the tree nests could have in fact been that of a gorilla. As a result, the difference between the ape and ‘chimpanzee’ abundance can be assumed to be the minimum population size for gorillas, which comes out to 3,273 individuals.
