# Supplementary material for: Priority Areas for Large Mammal Conservation in Equatorial Guinea
Source: PLoS One. 2013 Sep 27;8(9):e75024. doi: 10.1371/journal.pone.0075024 (PMC3785506; doi:10.1371/journal.pone.0075024)
Supplement: Text S2 — Autocorrelation. (DOC) [file pone.0075024.s010.doc]

**Text S2**. **Autocorrelation**

For calculating the autocorrelation term we first ran a model for every response variable that included all six predictors and derived residuals for every data point (transect). We then calculated for each transect the autocorrelation term by averaging the residuals of all other transects weighted by the Euclidean distance. The weight followed a normal distribution and we estimated the standard deviation by minimizing the AIC of the GLM including the calculated autocorrelation term as additional variable.
