# Supplementary material for: Priority Areas for Large Mammal Conservation in Equatorial Guinea
Source: PLoS One. 2013 Sep 27;8(9):e75024. doi: 10.1371/journal.pone.0075024 (PMC3785506; doi:10.1371/journal.pone.0075024)
Supplement: Text S3 — Comparison of design based and spatial model predictions. (DOC) [file pone.0075024.s011.doc]

**Text S3. Comparison of design based and spatial model predictions**

There was a clear correlation between predicted (non road model) and observed density at transect location (Spearman correlation, *r*_s_=0.57, *n*=83, *p*<0.01). Predicted density (0.43 ind/km²) was 9.8% higher as compared to observed density (0.39 ind/km²). This difference seems to be driven by a single transect for which a density of about 3.2 ape individuals was predicted, but on which only four nests were found (Figure S3). The removal of this transect from the comparison then yields a density of 0.390 and 0.397 ind/km² for observed and predicted density, respectively.
