# Supplementary material for: Priority Areas for Large Mammal Conservation in Equatorial Guinea
Source: PLoS One. 2013 Sep 27;8(9):e75024. doi: 10.1371/journal.pone.0075024 (PMC3785506; doi:10.1371/journal.pone.0075024)
Supplement: Text S4 — Comparison with previous estimates. (DOC) [file pone.0075024.s012.doc]

**Text S4. Comparison with previous estimates**

Even our minimum gorilla estimate is much higher than the previous estimate of 1,000-2,000 individuals following a nationwide gorilla census between 1989 and 1990 [32]. The long generation times of gorillas and bushmeat hunting offtakes make it unlikely that this difference is due to an increase in the population. Hence a more probable explanation for the difference in findings between the studies is likely due to the methodology making the data not directly comparable. The placement of line transects was different first of all, and the nest degradation rate also differed considerably. It is therefore not straightforward to estimate the change in their population with past studies.
